# The CPT1C 5′UTR Contains a Repressing Upstream Open Reading Frame That Is Regulated by Cellular Energy Availability and AMPK

**DOI:** 10.1371/journal.pone.0021486

**Published:** 2011-09-22

**Authors:** Ines Lohse, Patrick Reilly, Kathrin Zaugg

**Affiliations:** 1 Department of Radiation Oncology, University Hospital Zurich, Zurich, Switzerland; 2 Department of Cellular and Molecular Research, National Cancer Centre Singapore, Singapore, Singapore; Texas A&M University, United States of America

## Abstract

**Background:**

Translational control is utilized as a means of regulating gene expression in many species. In most cases, posttranscriptional regulatory mechanisms play an important role in stress response pathways and can lead to dysfunctional physiology if blocked by mutations. Carnitine Palmitoyltransferase 1 C (CPT1C), the brain-specific member of the CPT 1 family, has previously been shown to be involved in regulating metabolism in situations of energy surplus.

**Principal Findings:**

Sequence analysis of the CPT1C mRNA revealed that it contains an upstream open reading frame (uORF) in the 5′ UTR of its mRNA. Using CPT1C 5′ UTR/luciferase constructs, we investigated the role of the uORF in translational regulation. The results presented here show that translation from the CPT1C main open reading frame (mORF) is repressed by the presence of the uORF, that this repression is relieved in response to specific stress stimuli, namely glucose deprivation and palmitate-BSA treatment, and that AMPK inhibition can relieve this uORF-dependent repression.

**Significance:**

The fact that the mORF regulation is relieved in response to a specific set of stress stimuli rather than general stress response, hints at an involvement of CPT1C in cellular energy-sensing pathways and provides further evidence for a role of CPT1C in hypothalamic regulation of energy homeostasis.

## Introduction

The presence of upstream open reading frames (uORF) within mRNA 5′UTR can impact levels of translation initiation of the main open reading frame (mORF). Because eukaryotic ribosomes generally only initiate once per mRNA [1], the presence of an uORF normally inhibits the translation of the mORF and may lead to mRNA decay [2]–[8]. Although they are usually short sequences, uORFs may still play critical roles in modulating physiology. Indeed, mutations that introduce new or disrupt existing uORFs have been reported to cause human diseases [9]–[11].

Carnitine Palmitoyltransferase 1 (CPT1) C is a gene of the CPT1 family that is expressed specifically in the brain under normal conditions [12]. It is well established that CPT1A and CPT1B, the other two CPT1 family members, catalyze the initiating step of fatty acid degradation through which long-chain fatty acids (LCFA) are transported from the cytoplasm to the mitochondrial matrix for β-oxidation [13], [14]. In this enzymatic reaction, the fatty acyl group is transferred from acyl-CoA to carnitine to allow transport into mitochondria. The fact that CPT1C is mainly expressed in the CNS [12], [15], a tissue normally not using fatty acids (FA) as a major energy source, suggests a potentially unique function for CPT1C. Recent publications show that CPT1C expression in the brain is mainly restricted to the hypothalamic feeding centres, where lipid metabolism is believed to play a key role in regulating peripheral energy homeostasis. Results derived from studies using Cpt1c knock-out mice implicate CPT1C in the regulation of energy homeostasis and the control of food intake [16], [17]. The mechanisms by which CPT1C inhibition regulates feeding behavior remain elusive. It has been established that CPT1C, like the other CPT1 family members, binds LCFA, although the enzymatic activity of the CPT1C transferase domain is still controversial in the literature [15], [17]–[19]. Intracellular accumulation of saturated LCFA, for example palmitate, in non-adipose tissue leads to an inhibition of proliferation and apoptosis [20]–[22].

The maintenance of whole body energy homeostasis is critical for survival. This requires the presence of sensors that detect changes in whole body energy expenditure and induce adaptive responses. Hypothalamic feeding centers have been shown to regulate the desire for food intake and satiety and play an important role in the nervous system control of energy homeostasis [15], [23]–[27]. Hypothalamic cyclic-AMP dependent protein kinase (AMPK) activity is tightly regulated under physiological conditions and has been shown to play an important role in the hypothalamic regulation of energy homeostasis. AMPK is a heterotrimeric kinase complex composed of a catalytic α subunit and two regularly β and γ subunits. AMPK activity is enhanced by AMP binding and phosphorylation of the catalytic subunit by upstream kinases. Hypothalamic AMPK is responsive to alteration in cellular energy level, circulating hormones and nutritional cues. The modulation of AMPK activity in response to these factors initiates signaling pathways leading to changes in feeding behavior. These results show that a reduction in hypothalamic AMPK activity is sufficient to reduce food intake and body weight. In contrast, injection of the pharmacological AMPK agonist AICAR or the expression of constitutively active AMPK cause an increase in food intake and body weight. The molecular mechanism(s) by which AMPK regulates nutritional satiety involving the hypothalamus are still largely unknown but it may function through controlling differential hypothalamus gene expression [25], [28].

FAs are also known to be involved in the nervous control of energy homeostasis. It has been shown that free FAs are not used as fuel for neurons but serve as informative molecules about the whole body energy homeostasis. Specialized neurons within the hypothalamus detect variations of FA levels in the plasma and integrate this information in the regulation of peripheral glucose and lipid metabolism. Inhibition of the hypothalamic CPT1 by chemical inhibitors or the accumulation of certain LCFAs in the hypothalamus causes a reduction of food intake It has been hypothesized that high FA levels might serve as a signal for sufficient feeding and thereby reduce food intake [27], [29]–[34].

Here, we describe the existence of a conserved uORF element in the 5′UTR of CPT1C as well as its regulation by cellular energy sensing mechanisms. Analysis of the mRNA sequences revealed that CPT1C is the only CPT1 member that contains an uORF and that the presence of uORFs for Cpt1c is conserved in several mammalian species. Experiments using luciferase reporter constructs transfected into brain-derived cell lines reveal that the presence of the uORF inhibits the translation of the downstream mORF. In addition, these experiments show that the translation inhibition by the uORF is relieved in situations of glucose deprivation and palmitate-BSA treatment. Finally, we demonstrate by pharmacological and genetic interference, how AMPK activity controls this translation repression of CPT1C.

## Results

### Human *CPT1C* mRNA contains an uORF upstream of the mORF

The human *CPT1C* gene consists of 21 exons that give rise to two transcript variants (transcript variant 1 [NM_001136052], transcript variant 2 [NM_152359]). Although both mRNAs are transcribed from the same gene, both transcript variants differ in lengths and exon content. In both transcript variants exon 2 and parts of exon 16, and in transcript variant 2 also exon 3, are not part of the spliced mRNA. These exons, although not part of the mRNA might, however, have a regulatory function for either transcriptional or posttranscriptional regulation mechanisms.

Both mRNAs contain one uORF maintaining the same start codon but stop codons derived from exon 3 and exon 4 respectively. The uORF within transcript variant 1 starts at bp 97 and has a stop codon at bp 198 resulting in a 34 AA long peptide. The uORF within transcript variant 2 on the other hand starts at bp 91 and the corresponding stop codon at bp 206 resulting in a 37 AA long peptide. Neither of the peptides produced by the uORFs has any similarity to known proteins or protein domains.

### *CPT1C* is the only *CPT1* family member containing an uORF upstream of the Morf

*CPT1C* mRNA sequences of various species listed on NCBI showed that all mRNAs listed contain long 5′UTRs harbouring one or several uORFs (Table 1). The sequences and number of the mammalian *CPT1C* uORFs can vary significantly suggesting that the subsequent peptide(s) are not functional. For example, whereas the human *CPT1C* mRNA contains a single uORF out of frame with the mORF, the rat *Cpt1c* mRNA contains three uORF's that are located both in frame as well as out of frame with the mORF. Sequence analysis of resulting peptides from these uORF's from different species did not reveal any conserved elements except in the closely related primate species, suggesting that the peptide encoded does not have a conserved function. It should be noted that no orthologues of *CPT1C* have been annotated as such for non-mammalian vertebrates. To determine whether the presence of an uORF is also a common feature of the whole *CPT1* family, we analyzed the mRNA sequences of the other two *CPT1* family members, *CPT1A* and *CPT1B*. This analysis, however, revealed that both mRNAs have short 5′ UTRs containing no similar uORFs (Table 1). Indeed no annotated mammalian *CPT1A* or *CPT1B* paralog contain an uORF. This conservation suggests the CPT1C uORFs may be functional components of the mRNA that can regulate translation of the mORF.

**Table 1:** uORF distribution and 5′UTR length throughout the CPT1 family.

species		Accession number	5′UTR length	no. uORF's
human	CPT1A	NM_001876	170 bp	0
human	CPT1B	NM_004377	84 bp	0
human	CPT1C transcript variant 1	NM_001136052	281 bp	1
human	CPT1C transcript variant 2	NM_152359	206 bp	1
mus musculus	cpt1c	NM_153679	194 bp	2
rattus norwegicus	cpt1c	NM_001034925	199 bp	3
bos taurus	cpt1c	XM_002695120	225 bp	2
pogo abelii	predicted cpt1c	NM_001132026	228 bp	1

### Effects of the CPT1C uORF on the translation of the downstream mORF

**Figure 1:**
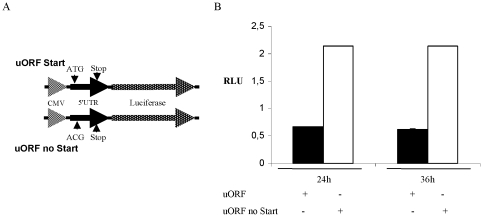
CPT1C 5′UTR induces reporter gene repression. (A) Reporter gene constructs containing the CPT1C 5′UTR cloned in front of a luciferase reporter gene with either the endogenous uORF start codon (ATG) or the mutated uORF start codon (ACG). (B) Luciferase activities of uORF start (filled bars) and uORF no start (empty bars) reporters in T98G cells after transfection and maintenance in normal conditions for 24 h or 36 h. *RLU*, Relative Luciferase Units.

Short uORFs can function as regulatory elements on the level of translation [2], [3], [4]. The presence of the uORF in both CPT1C transcript variants suggests that both variants can be regulated by the same mechanism. Because of this similarity between the transcript variants, we chose to only use the 5′UTR of CPT1C transcript variant 1 for further experiments. To analyse the uORF function, we cloned the *CPT1C* 5′UTR upstream of a luciferase reporter from a heterologous CMV immediate early promoter. As a negative control for non-uORF activities of the 5′UTR, we also cloned the same sequence where the start codon ATG of the uORF was mutated to ACG (Fig. 1A). Although the point mutation of the uORF start codon should not influence mRNA transcription or translation, we cannot fully exclude such effects. In order to exclude that the insertion of the CPT1C 5′UTR or the applied treadments influnce transcription or translation of the Luciferase reporter gene we used the original vector was used as a control in every experiment (data not shown). Transfection of glioblastoma-derived T98G cells demonstrated that the 5′UTR containing the mutated start codon exhibit a 3-fold higher luciferase activity than cells transfected with the 5′UTR containing the start codon (Fig. 1B). SV40-FHAS and U87-MG cells transfected with the plasmids containing the mutated uORF also showed an increase in luciferase activity (2.5- and 2.6-fold, respectively) when compared to the wild type (wt) luciferase reporter construct (Fig. S1). The difference in translation efficiency was observed 24 h as well as 36 h after transfection. These data indicate that the presence of the intact uORF of the *CPT1C* mRNA inhibits the translation of the mORF under normal growth conditions.

### Translation downstream of the *CPT1C* uORF is derepressed by glucose deprivation

Most of the reported uORFs have been shown to regulate the translation of the mORF in response to the cellular environment [2], [5], [6]. Since CPT proteins are predicted to function in regulating energy availability we chose to examine whether different metabolic stress stimuli influenced *CPTIC* 5′UTR activity. We examined expression from our uORF luciferase reporter constructs with altered cellular environments in T98G, SV40-FHAS and U87-MG cells.

**Figure 2:**
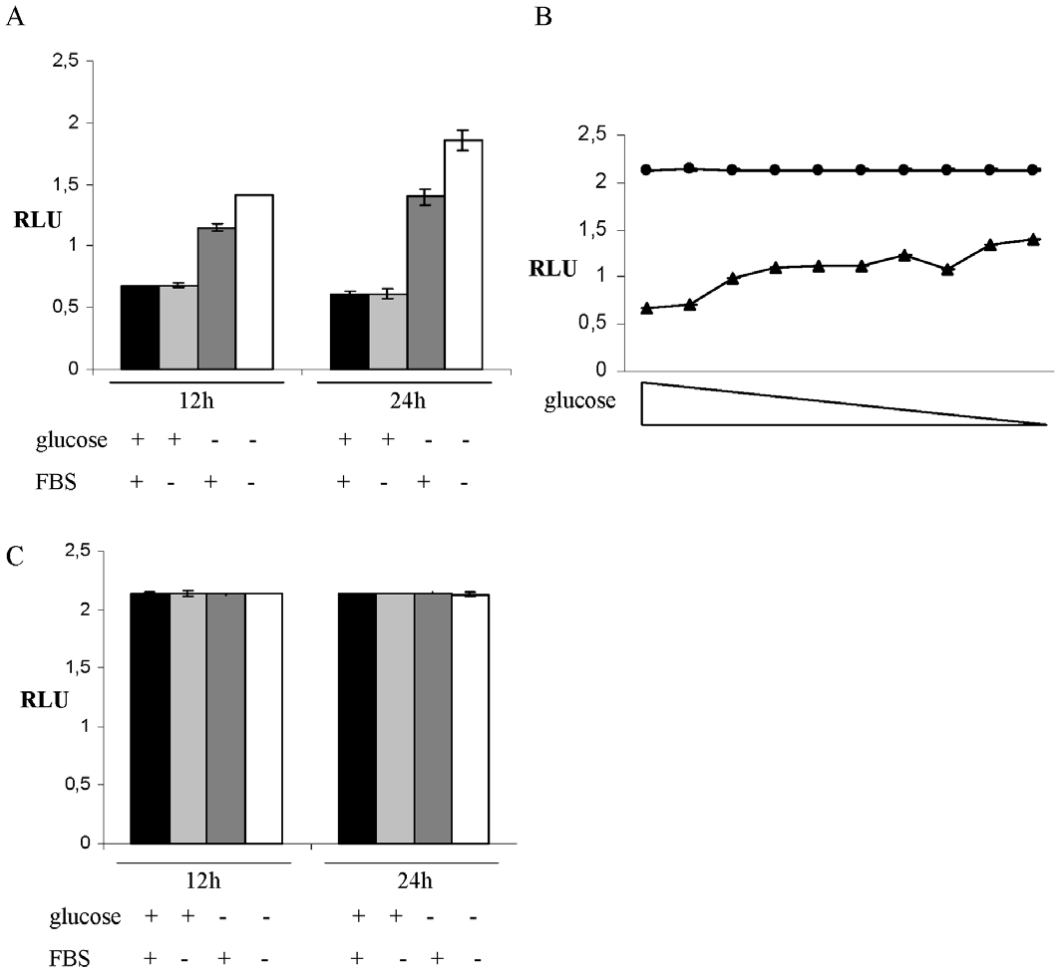
Modulation of CPT1C 5′UTR repression by varied growth conditions. (A) Relative luciferase activities of T98G cells transfected with the wt uORF reporter gene construct after glucose and/or serum deprivation for 12 h or 24 h. (B) Relative luciferase activities of T98G cells transfected with the wt uORF reporter gene construct (triangle) or mt uORF reporter gene construct (circle) with titration of glucose concentration in media after 12 hours. (C) Relative luciferase activities of T98G cells transfected with the no start uORF reporter gene construct after glucose and/or serum deprivation for 12 h or 24 h. *RLU*, Relative Luciferase Units.

To examine the influence of nutrient limitation on the uORF-mediated translational repression, the cells were incubated in media lacking glucose and/or FBS. In T98G cells the luciferase activity was increased by 1.5 fold after 12 h and by 2-fold after 24 h glucose deprivation (Fig. 2A) compared to cells grown in normal growth medium. Alone, FBS deprivation had no effect on luciferase activity at either time point (Fig. 2A). In the absence of glucose, however, FBS deprivation lead to an increase of 2-fold after 12 and 3-fold after 24 h treatment in T98G cells (Fig. 2A) when compared to cells grown in normal growth medium. The change of luciferase activity in SV40-FHAS and U87-MG cells upon treatment with the described deprivation conditions were similar to the changes observed in T98G cells (Fig. S2). To exclude that increased luciferase activity in response to glucose deprivation was derived from treatment-induced changes in transcriptional activity, cells transfected with the mutated 5′UTR construct were treated in parallel to the cells transfected with the wt 5′UTR constructed. In the absence of the intact ATG, we did not see any changes in luciferase activity in response to the deprivation media (Fig. 2C), suggesting that the derepression is not transcriptionally regulated but, rather, dependent on the uORF.

To elucidate at what glucose concentration the *CPT1C* 5′UTR repression is lost, we performed a glucose titration on transfected T98G cells. Because we saw little effect from the absence of FBS, we performed a glucose titration in the presence of FBS. The first strong increase in firefly luciferase activity can be observed at the titration step containing 0.62 µM of glucose with a stepwise increase of activity until the highest activity is reached in the medium containing no glucose. The general trend of slow increase in luciferase expression, however, suggests that glucose responsiveness of the *CPT1C* 5′UTR is not a tight switch but rather a concentration sensor of glucose or some glucose-dependent metabolite(s).

To exclude that the *CPT1C* 5′UTR is derepressed in response to general stress pathway activation rather than mediating a specific stress response, the cells were treated with 0.2% O_2_ for 12 h with or without a 12 h reoxigenation period following the hypoxic conditions. None of the tested hypoxia or reoxigenation conditions led to changes in reporter activity in T98G cells (Fig. S3).

### CPT1C uORF regulation is influenced by AMPK activity

To test whether derepression of the *CPT1C* 5′UTR in response to the cellular environment is dependent on the activation of AMPK, the transfected cells were treated with chemical AMPK activators and inhibitors. Additionally, we used the mTOR inhibitor Rapamycin to investigate a potential involvement of mTOR in potential uORF regulation.

**Figure 3:**
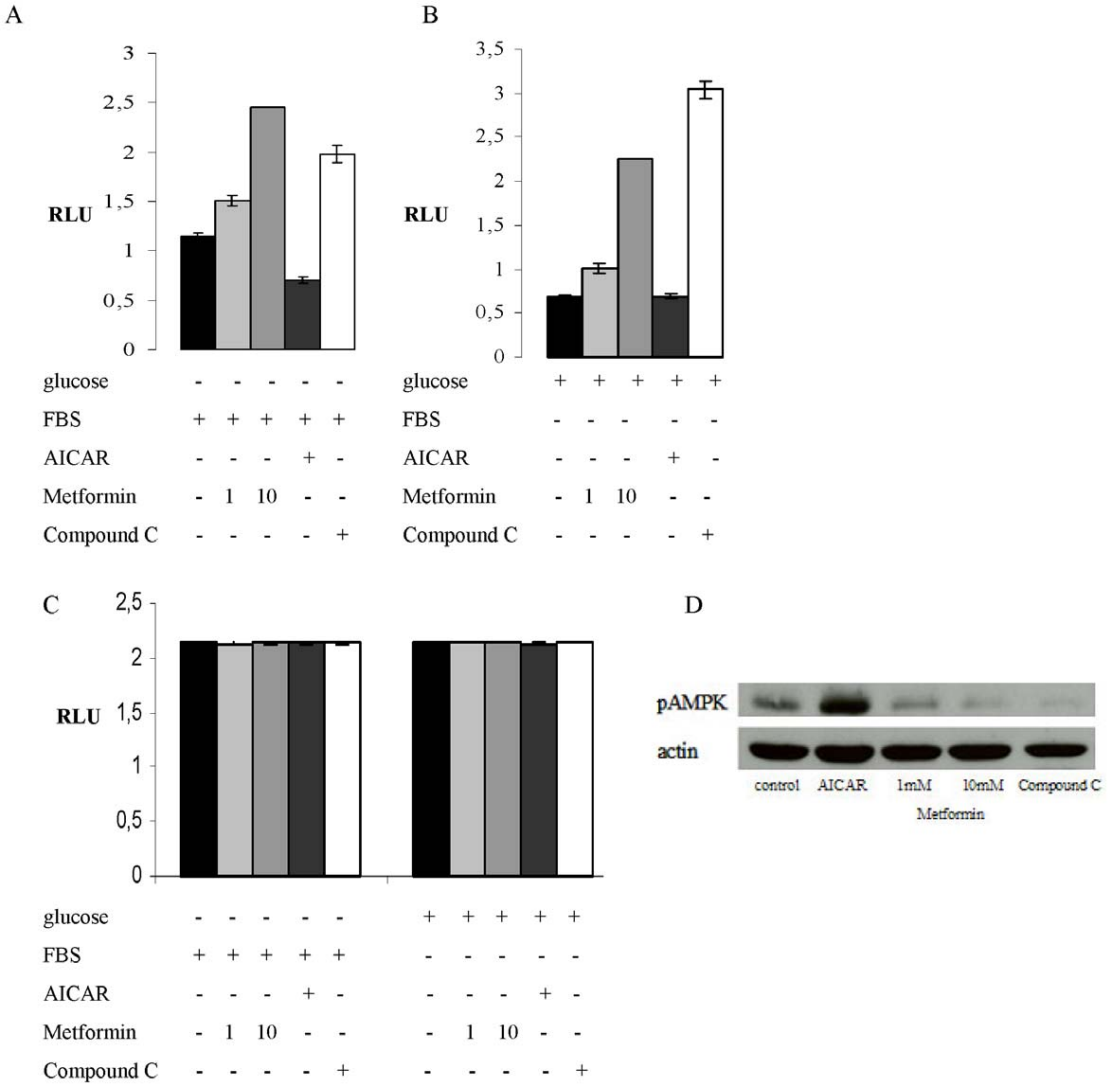
CPT1C 5′UTR repression is relieved by AMPK inhibition. Relative luciferase activity in T98G cells transfected with uORF start reporter construct after maintenance in media containing either (A) FBS but no glucose or (B) glucose but no FBS in the presence of AMPK-inhibitors metformin or Compound C, or in the presence of the AMPK-activator AICAR. (C) Relative luciferase activity in T98G cells and transfected with uORF no start reporter construct after maintenance in the presence of AMPK-activators metformin or Compound C or the AMPK-inhibitor AICAR. (D) AMPK phosphorylation status in T98G cells maintained in medium containing FBS but no glucose in the presence of AMPK-activators metformin or Compound C or the AMPK-inhibitor AICAR. *RLU*, Relative Luciferase Units.

To evaluate the effect of the chemical AMPK inhibitors Metformin and Compound C as well as the agonist AICAR, T98G cells maintained in glucose deprivation medium were treated with the chemical compounds for 6 h. Western Blot analysis revealed the expected increase in AMPK phosphorylation in response to AICAR treatment (Fig. 3D). Whereas Metformin is commonly classified as an AMPK agonist, in our study treatment with Metformin resulted in a dose dependent decrease in AMPK phosphorylation that was also observed in the samples treated with Compound C (Fig. 3D). Such metformin inhibition of AMPK in neuronal cells has been previously reported [35]–[38]. Treatment with metformin, with and without glucose deprivation led to an increase in luciferase activity in a dose-dependent manner. T98G cells treated with 1 mM or 10 mM Metformin showed a 1.2- or 2-fold increase, respectively, in reporter activity under glucose deprivation conditions and a 1.6- or 3.6-fold increase, respectively, if treated with FBS deprivation (Fig. 3B). Consistent with AMPK positively activating the *CPT1C* 5′UTR repression mechanism, treatment with the AMP agonist AICAR [21], [35]–[43] decreased reporter activity under glucose deprivation conditions, but not in FBS deprivation conditions (Fig. 3B).

The AMPK-specific inhibitor, Compound C [35]–[43], also induced derepression from the 5′UTR, consistent with that seen for metformin. Curiously, with this compound, higher derepression of the reporter activity was seen in the FBS deprivation than in glucose deprivation conditions. The increase in luciferase activity was a 4.9-fold increase in FBS deprivation, if compared to a 1.7-fold increase in cells treated with glucose deprivation (Fig. 3B). We suggest that this effect is likely due to different pharmacodynamics of Compound C in the presence of serum. Again, similar results were seen with the alternate cell line SV40-FHAS (Fig. S4). Analogous to the results shown in Figure 3C, treatment with the pharmacological inhibitors in combination with the deprivation media did not affect the luciferase activity in cells transfected with the mutated 5′UTR construct containing no Start codon (Fig. 3C). Taken together, this data indicates that AMPK activity likely plays an important role in regulating this translational repression from the *CPT1C* 5′UTR.

Inhibition of mTOR by Rapamycin did not influence the *CPT1C* 5′UTR in either of the tested cell lines under either of the deprivation conditions (Fig. S3). This suggest that the CPT1C 5′UTR is responsive to energy supply and not responsive to stimuli that signal through mTOR.

### *CPT1C* uORF repression is disrupted by AMPK depletion

**Figure 4:**
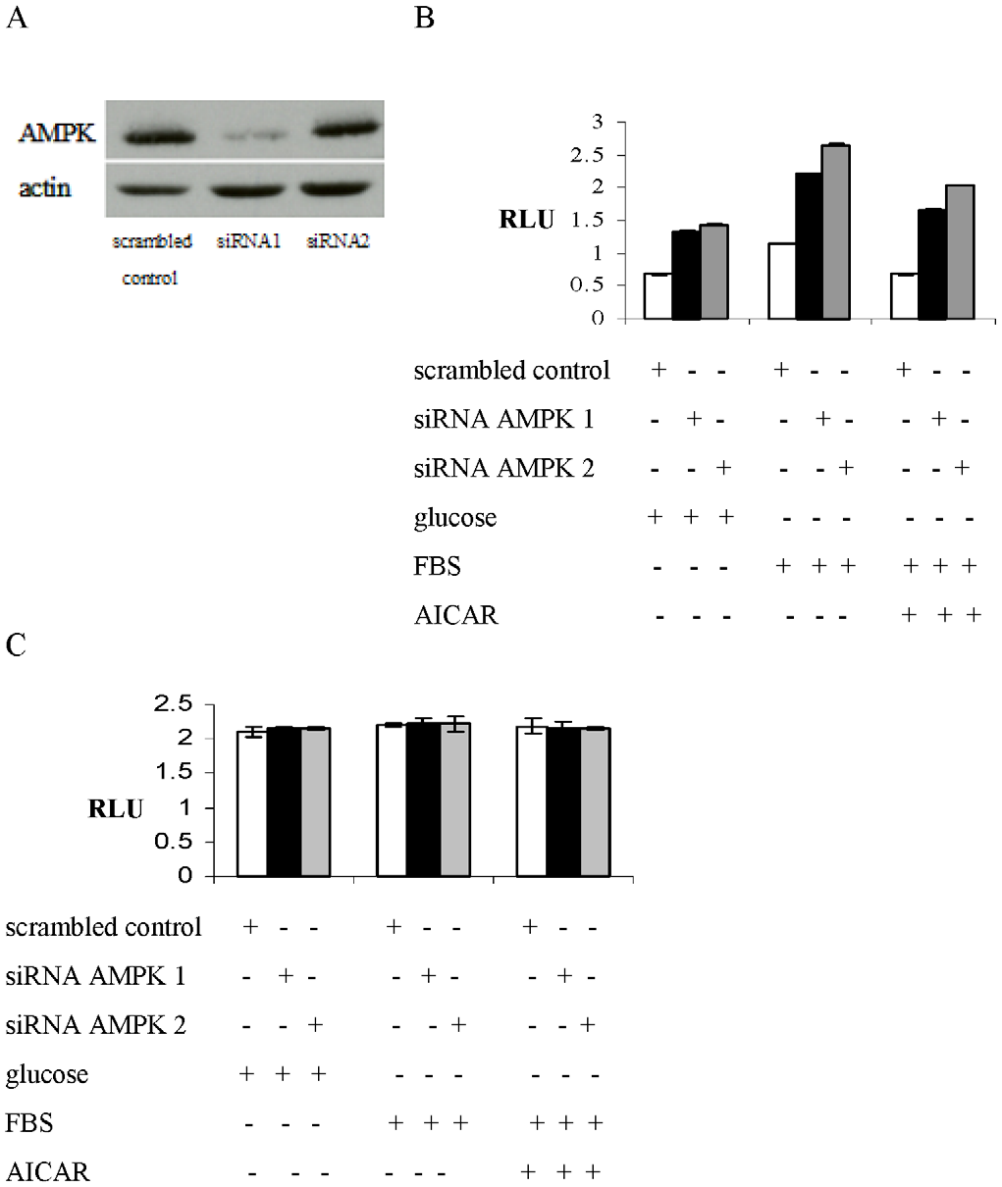
CPT1C 5′UTR repression is relieved by AMPK knock down. (A) AMPK mRNA expression in T98G cells treated with either the control siRNA or the siRNA targeting AMPK maintained in medium containing FBS. (B) Relative luciferase activity in siRNA treated T98G cells transfected with uORF start reporter construct after maintenance in media containing either glucose but no FBS or FBS but no glucose or FBS but no glucose in the presence of the AMPK-activator AICAR. (C) Relative luciferase activity in siRNA treated T98G cells and transfected with uORF no start reporter construct after maintenance in media containing either glucose but no FBS or FBS but no glucose or FBS but no glucose in the presence of the AMPK-activator AICAR. *RLU*, Relative Luciferase Units.

To test whether the derepression of the reporter activity in response to the treatment with the chemical AMPK inhibitors depend on AMPK activity, T98G cells were treated with either an unspecific control siRNA or siRNAs targeted against AMPKα1 (Table S1). The knock down of AMPKα1 was evaluated 48 h after transfection in cells maintained in glucose deprivation medium (Fig. 4A). T98G cell transfected with the 5′UTR construct 48 h after siRNA transfection revealed a derepression of reporter activity in response to AMPK knockdown (Fig. 4B). The increase in luciferase activity was similar to that observed after treatment with the chemical AMPK inhibitors and was not reduced in response to treatment with the AMPK agonist AICAR. Analogous to the results shown in Figure 2C and 3C, siRNA transfection did not affect the luciferase activity in cells transfected with the mutated 5′UTR construct (Fig. 4C). As shown in Figure 4A the two siRNAs used show a different knock down efficency towards AMPK alpha 1 protein level. In the Luciferase reporter assay, however, are similarly effective in derepressing the CPT1C ORF (Fig. 4B). We hypothesize that a reduction od AMPK alpha 1 beyond a certain threshold will ultimativly lead to a derepression of CPT1C ORF translation.

### Palmitate-BSA treatment releases the CPT1C mORF from the uORF induced repression

**Figure 5:**
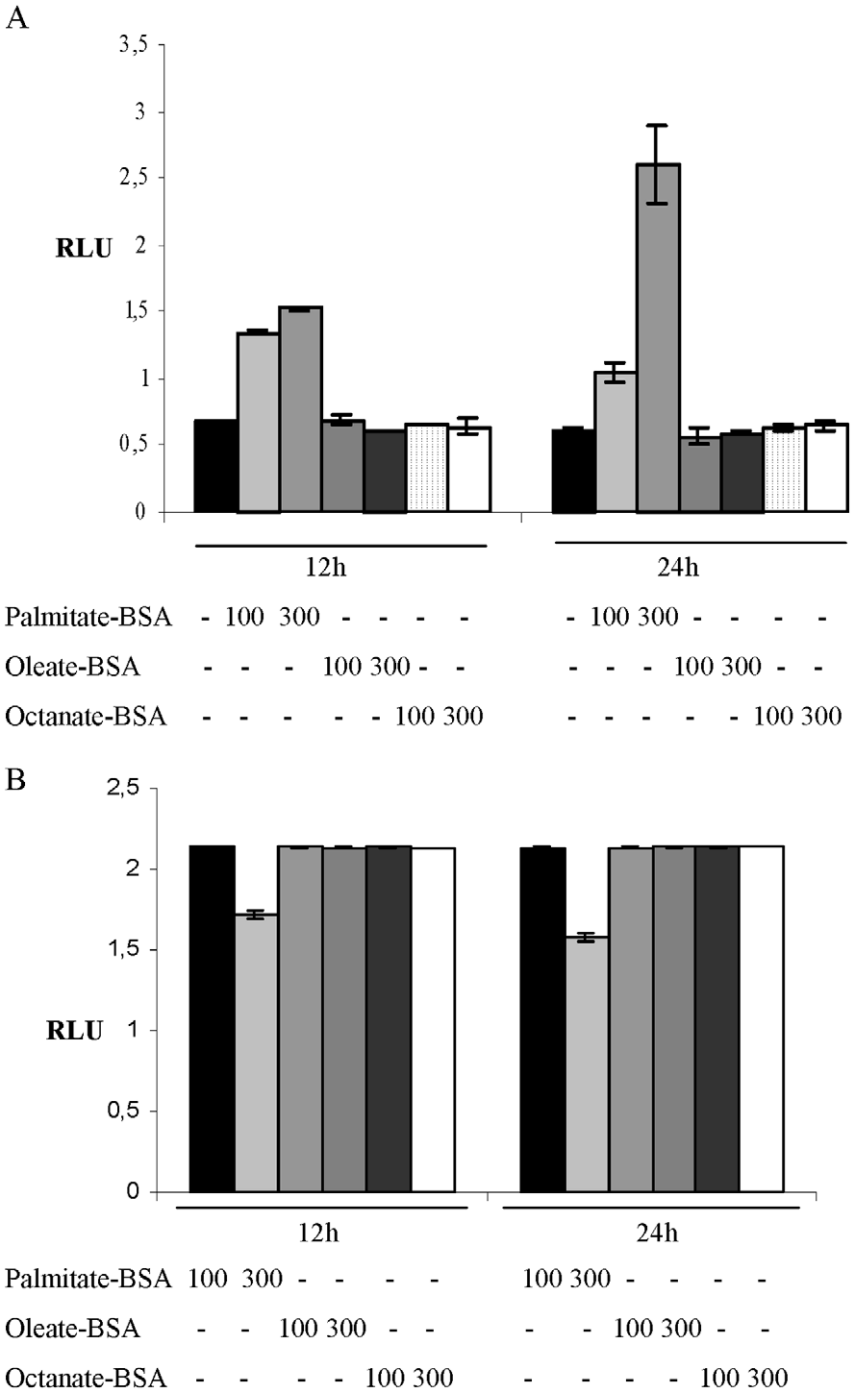
Palmitate relieves CPT1C 5′UTR-mediated translational repression. Relative luciferase activity of (A) uORF start and (B) uORF no start reporter constructs after transfection into T98G cells upon treatment with stated BSA conjugated fatty acids for 12 h or 24 h. *RLU*, Relative luciferase units, *Palmitate-BSA*, Palmitic acid conjugated BSA, *Oleate-BSA*, Oleic acid conjugated BSA, *Octanate-BSA*, Octanaic acid-conjugated BSA. *RLU*, Relative Luciferase Units.

To evaluate the impact of palmitic acid, oleic acid and octanoic acid on the regulation of the mORF by the uORF, the transfectants were treated with either 100 µM or 300 µM FA-BSA. Palmitic and oleic acid are both LCFA and known substrates of CPT1A and CPT1B, whereas CPT1 protein does not catalyze import of short chain fatty acid, for example oleic acid, into the mitochondria [14], [26], [44]. In normal glucose conditions, treatment with 100 µM Palmitate-BSA for 12 h led to a 1.9-fold increase in luciferase activity in T98G cells (Fig. 5A) and 2.6-fold in SV40-FHAS cells (Fig. S5A), if compared to the untreated control. Here, the increase in luciferase activity was not maintained over a treatment period of 24 h possibly due to substrate decomposition. After 12 h of treatment, 300 µM Palmitate-BSA led to a small further increase over 100 µM treatment levels. At this concentration, however, a further increase (5-fold above normal) could be seen after 24 h of treatment. This is equivalent to a 4-fold increase over 100 µM Palmitate-BSA treatment (Fig. 5A). Furthermore, SV40-FHAS cells exhibited an 8-fold increase over normal conditions after 24 h of treatment (Fig. S5B). Treatment with neither oleic or octanioc acid at the tested FA concentrations resulted in increased luciferase activity (Fig. 5A). In contrast to the results shown in Figure 2, 3 and 4 where none of the treatments did affect the luciferase activity in cells transfected with the mutated 5′UTR, treatment with 300 µM Palmitate-BSA did lead to a significant decrease of luciferase activity, which suggest a decrease of transcription in response to the treatment. This decrease was not observed when the cells where treated with 100 µM Palmitate-BSA or one of the other two FA at either concentration (Figure 5B). The reduction of transcriptional activity seen in the 300 µM Palmitate-BSA treated cells suggests that the translational derepression is stronger than indicated by the fold change in the wt 5′UTR transfected cells (Figure 4A). These data suggest that there is a concentration sensing and a time-dependent sensing mechanism used for derepression of the CPT1C 5′UTR.

### AMPK and Palmitate-BSA regulate CPT1C translation through independent pathways

Changes in whole-body energy status can be sensed via different nutrient-sensitive neuronal populations within the hypothalamic feeding centers that react to different sets of stimuli. In order to examine whether the cellular energy state by the means of AMPK activity and Palmitate-BSA regulate CPT1C translation via a common mechanism, T98G cells maintained in media either containing glucose but no FBS or FBS but no glucose were treated with the chemical AMPK inhibitors and the AMPK agonist in the presence of Palmitate-BSA.

**Figure 6:**
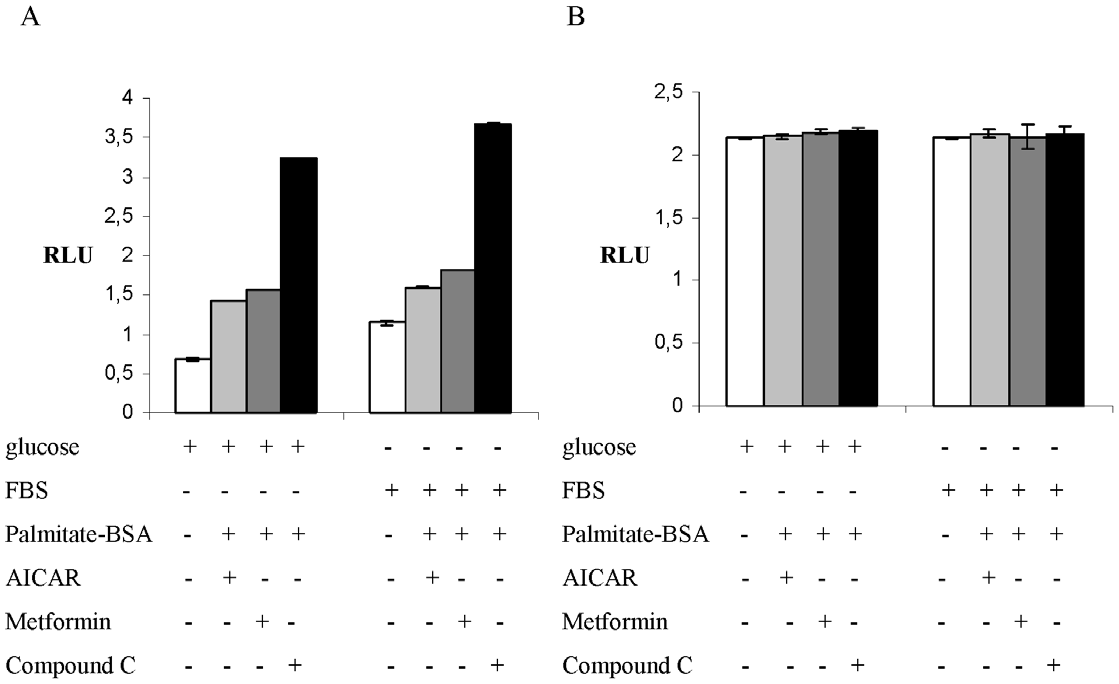
Palmitate and AMPK relieve CPT1C 5′UTR-mediated translational repression via different pathways. (A) Relative luciferase activity in T98G cells transfected with uORF start reporter construct after maintenance in media containing either glucose but no FBS or FBS but no glucose or FBS but no glucose supplemented with the AMPK-inhibitor AICAR. (B) Relative luciferase activity in T98G cells and transfected with uORF no start reporter construct after maintenance in media either containing glucose but no FBS or FBS but no glucose or FBS but no glucose supplemented with the AMPK-activator AICAR. *RLU*, Relative Luciferase Units.

Treatment with Metformin or Compound C combined with Palmitate-BSA, with or without glucose deprivation led to an additive increase in luciferase activity (Fig. 6A) if compared to the inhibitor treatment alone (Fig. 3A, B). Treatment with AICAR in the presence of Palmitate-BSA did not reduce luciferase expression in cells maintained in glucose deprivation media (Fig. 6A) as it was observed in cells treated with AICAR alone (Fig. 3A). Analogous to the results shown in Figure 2, 3 and 4, the treatment did not affect the luciferase activity in cells transfected with the mutated 5′UTR construct (Fig. 6B). These data suggest that AMPK and FA may regulate CPT1C translational initiation via independent mechanisms. Alternately, AMPK, in response to low glucose, may mobilize FA to ultimately regulate this translational repression.

## Discussion

The above data suggest that CPT1C can be regulated through an uORF and that this regulation can be impacted by cellular energy availability and AMPK activity. This regulation would describe a heretofore unseen mechanism for metabolic control of gene expression. Whereas we would propose that such a mechanism would have a very limited range of targets, the rate-limiting role of CPT proteins in FA metabolism would suggest that it could be physiologically important.

The enzymatic activity of CPT1C remains enigmatic. Although CPT1C has structural similarities to the other CPT1 isoforms and binds malonyl-CoA, neither its enzymatic activity nor its substrate(s) are established [15], [17]–[19]. Indeed the localization of CPT1C is also controversial, as it has been localized to both the mitochondria and ER in different studies.

Together with recent evidence for transcriptional control of CPT1C in the hypothalamus [33], our finding that translational mechanisms are used to control CPT1C suggests that CPT1C expression needs to be tightly regulated in the hypothalamus. Our results indicate that uORF regulation within the 5′UTR maintains low basal CPT1C expression during unstressed conditions and is important in the translational induction for expression during reduced energy availability. Regulation at a transcriptional level would suggest that CPT1C protein levels must rapidly change to fulfil its role.

Combined with the fact that the Cpt1c gene-deficient mouse demonstrates a metabolic phenotype [15], there is increasing evidence that CPT1C gene expression may be an important effector in regulating satiety. Here we provide evidence for a potential means of regulation of CPT1C by AMPK, a suspected regulator of satiety. Thus, we offer a novel potential mechanism for AMPK function in control of satiety. The factors downstream of CPT1C that may control satiety remain to be elucidated.

Our studies of combining the individual derepressive stimuli would suggest either these AMPK and FA may act in converging pathways for CPT1C uORF derepression, or act in a defined pathway whereby low carbohydrate concentration may signals AMPK, as is reported. AMPK may then, in turn, mobilize FA that can then act to derepress the CPT1C uORF. Further biochemical studies will be necessary to elucidate the specific mechanism through which Palmitate and AMPK activation may control this translational derepression.

## Materials and Methods

### Plasmid construction

The 5′UTR of the CPT1C transcript was amplified from the cDNA clone (MGC, Invitrogen) by PCR under standard conditions using the following primer (restriction sites are underlined) fwd 5′-CTC GAG GGA ATC GGG GTT TCT GGG TGA CGG-3′and rev 5′-AAG CTT GTC ACG CTG GAG CCC ACG and cloned into a TA vector (Invitrogen). To create a construct lacking a functional start codon in addition to the wt 5′UTR construct, the ATG of the uORF was mutated to ACG with the following primers fwd 5′-GGC ATT GGA CAT ACG CAA GCG GGA G-3′ and rev 5′-CTC CCG CTT GCG TAT GTC CAA TGC C-3′ using a standard side directed mutagenesis PCR. After sequencing, the XhoI/HindIII restricted inserts of both constructs were subcloned into the phCMV-Cluc-FSR vector (Genlantis).

### Preparation of Fatty Acid-BSA complexes

20% essentially fatty acid free BSA was prepared in 150 mM NaCl and sterile filtered. 20 mM fatty acid was sapoficated and dissolved in 150 mM NaCl heated up to 65°C. The free fatty acid was complexed to BSA by adding an equal volume 20% BSA to the 65°C fatty acid solution and stirred for 10 min. The solution was allowed to cool down under stirring and sterile filtered before alliquoting.

### Cell culture

The human Glioblastoma cell lines T98G and U87-MG and the SV40 large T-Ag immortalized fetal human non-neoplastic astrocytic cell line SV40-FHAS were purchased from the American Type Culture Collection (ATCC). The cells were maintained in monolayer culture in DMEM containing 4.5 g of glucose supplemented with 10% FBS, 1% L-Glutamine and 1% Penicillin/Streptomycin. All cells were grown in a humidified atmosphere of 5% CO_2_ at 37°C. For hypoxic conditions, the T98G cells were cultured at 37°C with 5% CO_2_, 94% N_2_ and 0.2% O_2_ in a hypoxic incubator (Scholzen AG). For all fatty acid treatments the supplements were added to the normal growth medium. For the starving conditions either DMEM or DMEM without glucose supplemented with 1% L-Glutamine and 1% Penecillin/Streptomycin with and without 10% FBS was used. T98G cells were transfected with the siRNAs (Tab. S1) using Lipofectamine 2000 (Invitrogene) using standard protocols 48 h prior to the Luciferase assay.

### Luciferase assay

Cells were co-transfected with the firefly luciferase constructs and the pRL-SV40 renilla luciferase vector (Promega) using Lipofectamine 2000 (Invitrogene) and seeded 12 h prior to treatment with 10^4^ cells in 96 Micro-Assay-Plates (Greiner-bio-one). Relative luciferase levels were determined by the ratio of firefly luciferase and renilla luciferase activity. The luciferase activities were measured by the Dual-Glo Luciferase System (Promega) according to the Dual-Glo Luciferase System manual (Promega) using the Glomax 96-well luminometer (Promega). All experiments were repeated at least 3 times in triplicates and included Mock transfections and transfections with the phCMV-cLuc-FSR vector.

### Western Blot

Total protein was extracted after irradiation and protein concentration was determined. 50 µg per sample were separated by 8% SDS gel and transferred onto PVDF membrane (GE Healthcare). The membranes were blocked with 2% non-fat-dry milk and probed with the anti-protein antibodies (AMPKα, pAMPKα (Thr 172) [Cell Signalling Technology]; tubulin [SIGMA Aldrich], H5 [SIGMA-Aldrich]). The blots were further incubated with horse radish peroxidase (HRP)-labeled antibodies (GE Healthcare) and the specific complexes were detected using the ECL Western Blotting Detection Reagents (GE Healthcare).

## Supporting Information

Figure S1 **CPT1C 5′UTR induces reporter gene repression.** Luciferase activities of uORF start (filled bars) and uORF no start (empty bars) reporters in (A) SV40-FHAS cells or (B) U87-MG cells after transfection and maintenance in normal conditions for 24 h or 36 h. *RLU*, Relative Luciferase Units.
(TIF)

Figure S2 **Modulation of CPT1C 5′UTR repression by varied growth conditions.** Relative luciferase activities of (A, B) SV40-FHAS cells or (C, D) U87-MG cells transfected with the wt uORF reporter gene construct after (A, C) glucose and/or serum deprivation for 12 h or 24 h. (B, D) Relative luciferase activities of SV40-FHAS cells transfected with the no start uORF reporter gene construct after glucose and/or serum deprivation for 12 h or 24 h. *RLU*, Relative Luciferase Units.
(TIF)

Figure S3 **Modulation of CPT1C 5′UTR repression by hypoxia and rapamycin.** Relative luciferase activities of (A, B,C) T98G cells or (D, E) SV40-FHAS cells transfected with the (A; B, D) wt uORF reporter gene construct or with the (C, E) no start uORF reporter gene construct after (A) 12 h Hypoxia, (A) 12 h Hypoxia and 8 h Reoxigenation or (B, C, D, E) Rapamycin treatment. *RLU*, Relative Luciferase Units.
(TIF)

Figure S4 **CPT1C 5′UTR repression is relieved by AMPK inhibition.** Relative luciferase activity in SV40-FHAS cells transfected with uORF start reporter construct after maintenance in media containing either (A) FBS but no glucose or glucose but no FBS in the presence of AMPK-inhibitors metformin or Compound C, or in the presence of the AMPK-activator AICAR. (B) Relative luciferase activity in cells and transfected with uORF no start reporter construct after maintenance in the presence of AMPK-activators metformin or Compound C, the AMPK-inhibitor AICAR. *RLU*, Relative Luciferase Units.
(TIF)

Figure S5 **Palmitate relieves CPT1C 5′UTR-mediated translational repression.** Relative luciferase activity of (A) uORF start and (B) uORF no start reporter constructs after transfection into SV40-FHAS cells upon treatment with stated BSA conjugated fatty acids for 12 h or 24 h. RLU, Relative luciferase units, Palmitate-BSA, Palmitic acid conjugated BSA, Oleate-BSA, Oleic acid conjugated BSA, Octanate-BSA, Octanoic acid-conjugated BSA. *RLU*, Relative Luciferase Units.
(TIF)

Table S1 **siRNA sequences.** Sequences of the shRNAs used for transient AMPK knock down and the scarmbeled control shRNA that was used as a nonspecific control.
(DOC)
