# Supplementary figures and images for: The CPT1C 5′UTR Contains a Repressing Upstream Open Reading Frame That Is Regulated by Cellular Energy Availability and AMPK
Source: PLoS One. 2011 Sep 22;6(9):e21486. doi: 10.1371/journal.pone.0021486 (PMC3178533; doi:10.1371/journal.pone.0021486)

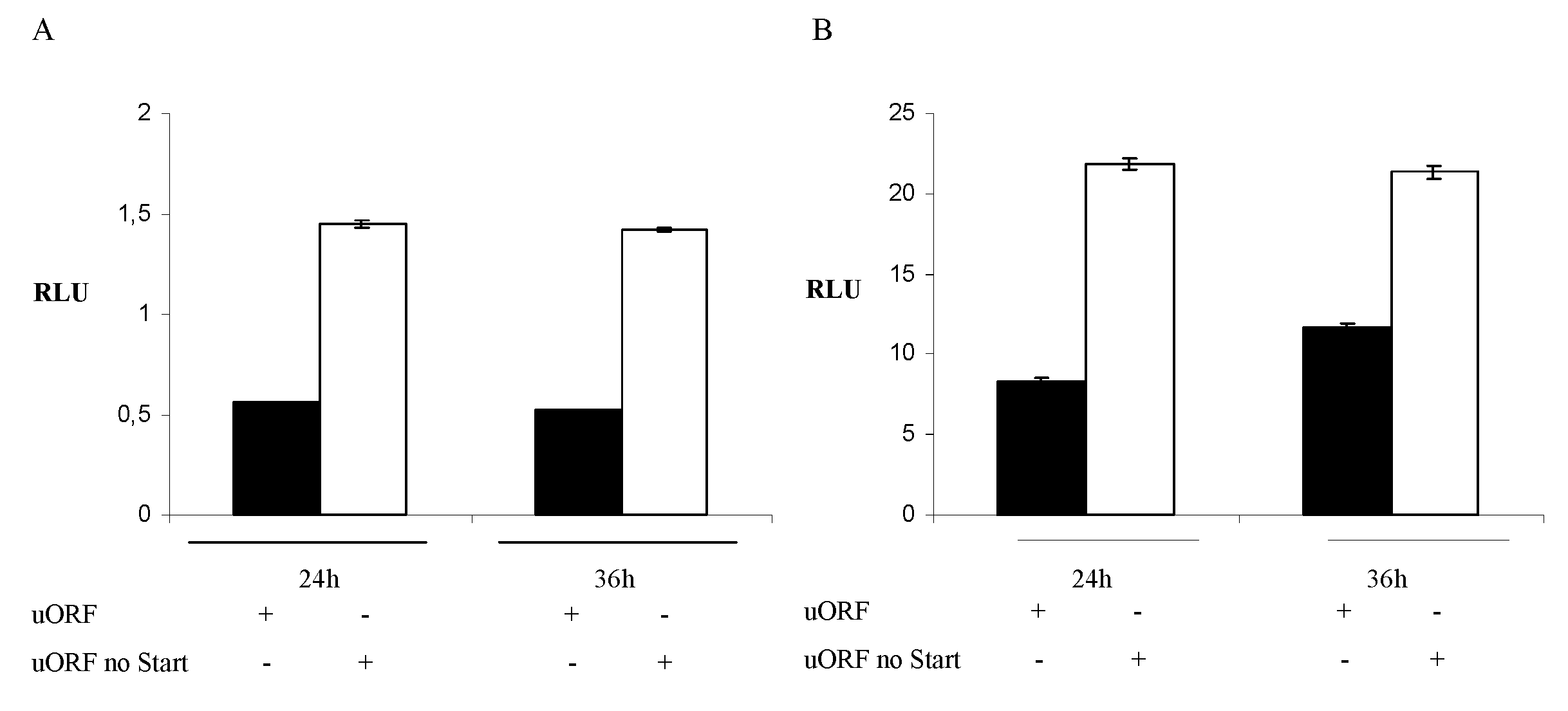

Supplement: Figure S1 — CPT1C 5′UTR induces reporter gene repression. Luciferase activities of uORF start (filled bars) and uORF no start (empty bars) reporters in (A) SV40-FHAS cells or (B) U87-MG cells after transfection and maintenance in normal conditions for 24 h or 36 h. RLU, Relative Luciferase Units. (TIF) [file pone.0021486.s001.tif]

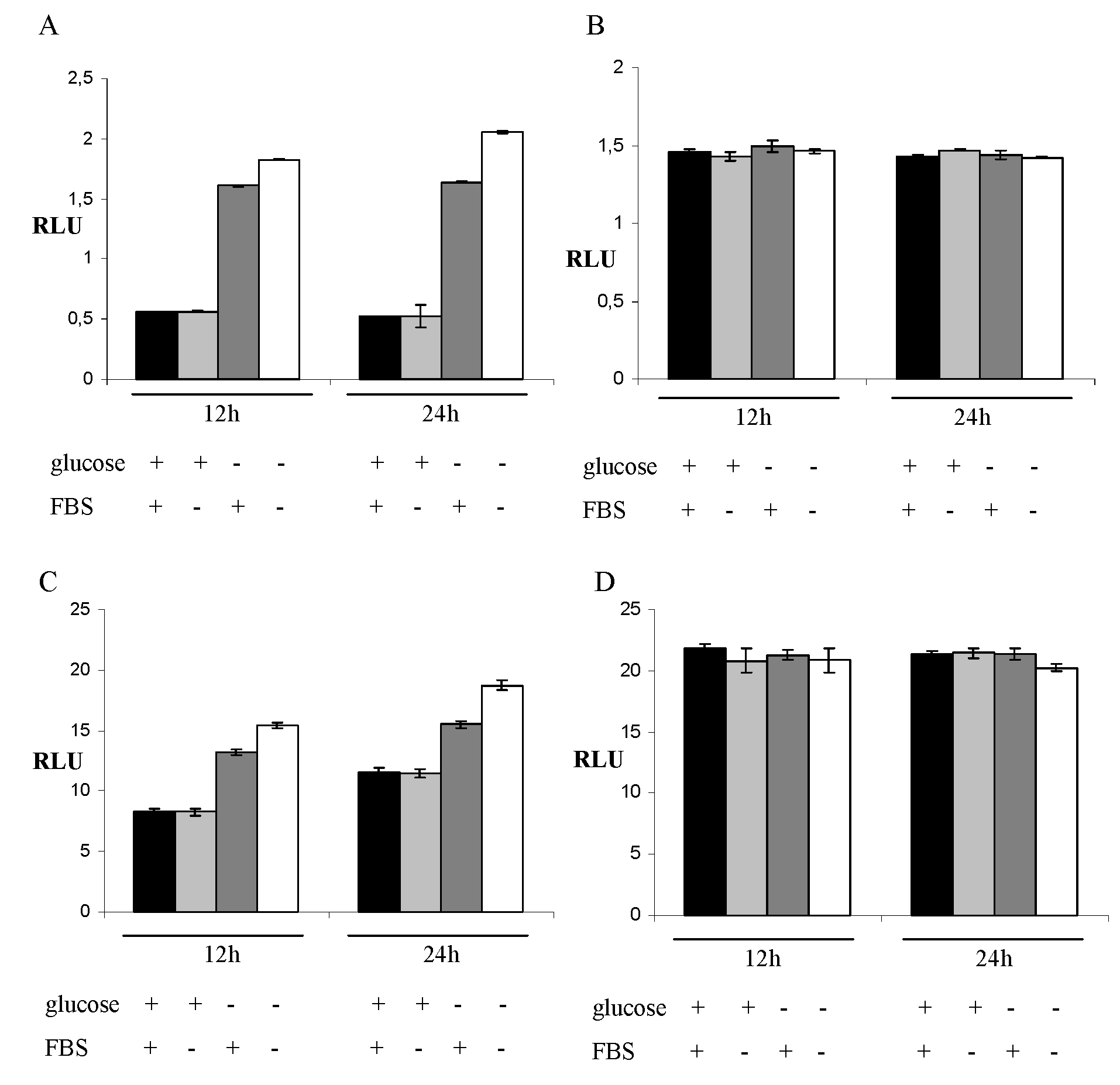

Supplement: Figure S2 — Modulation of CPT1C 5′UTR repression by varied growth conditions. Relative luciferase activities of (A, B) SV40-FHAS cells or (C, D) U87-MG cells transfected with the wt uORF reporter gene construct after (A, C) glucose and/or serum deprivation for 12 h or 24 h. (B, D) Relative luciferase activities of SV40-FHAS cells transfected with the no start uORF reporter gene construct after glucose and/or serum deprivation for 12 h or 24 h. RLU, Relative Luciferase Units. (TIF) [file pone.0021486.s002.tif]

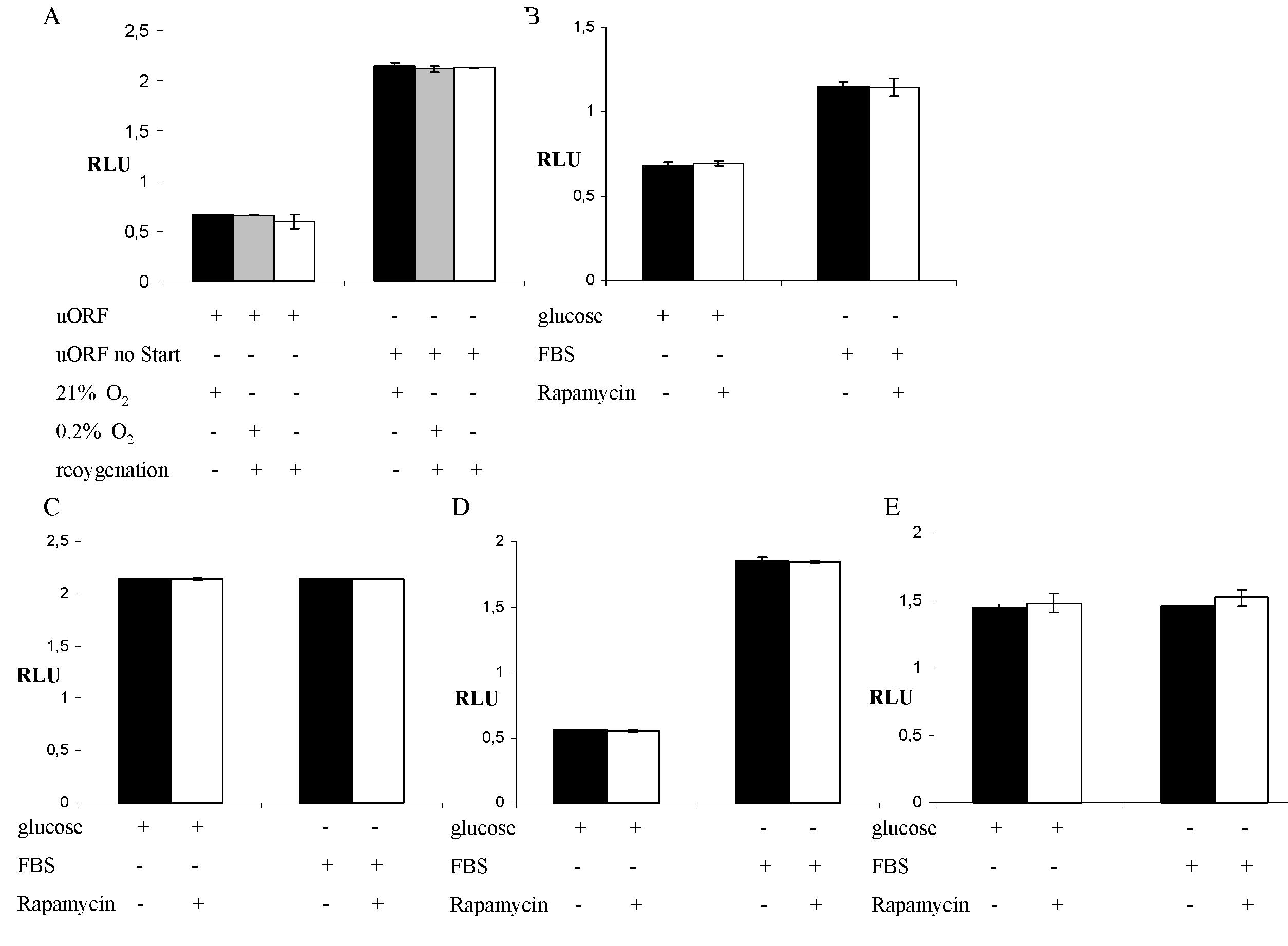

Supplement: Figure S3 — Modulation of CPT1C 5′UTR repression by hypoxia and rapamycin. Relative luciferase activities of (A, B,C) T98G cells or (D, E) SV40-FHAS cells transfected with the (A; B, D) wt uORF reporter gene construct or with the (C, E) no start uORF reporter gene construct after (A) 12 h Hypoxia, (A) 12 h Hypoxia and 8 h Reoxigenation or (B, C, D, E) Rapamycin treatment. RLU, Relative Luciferase Units. (TIF) [file pone.0021486.s003.tif]

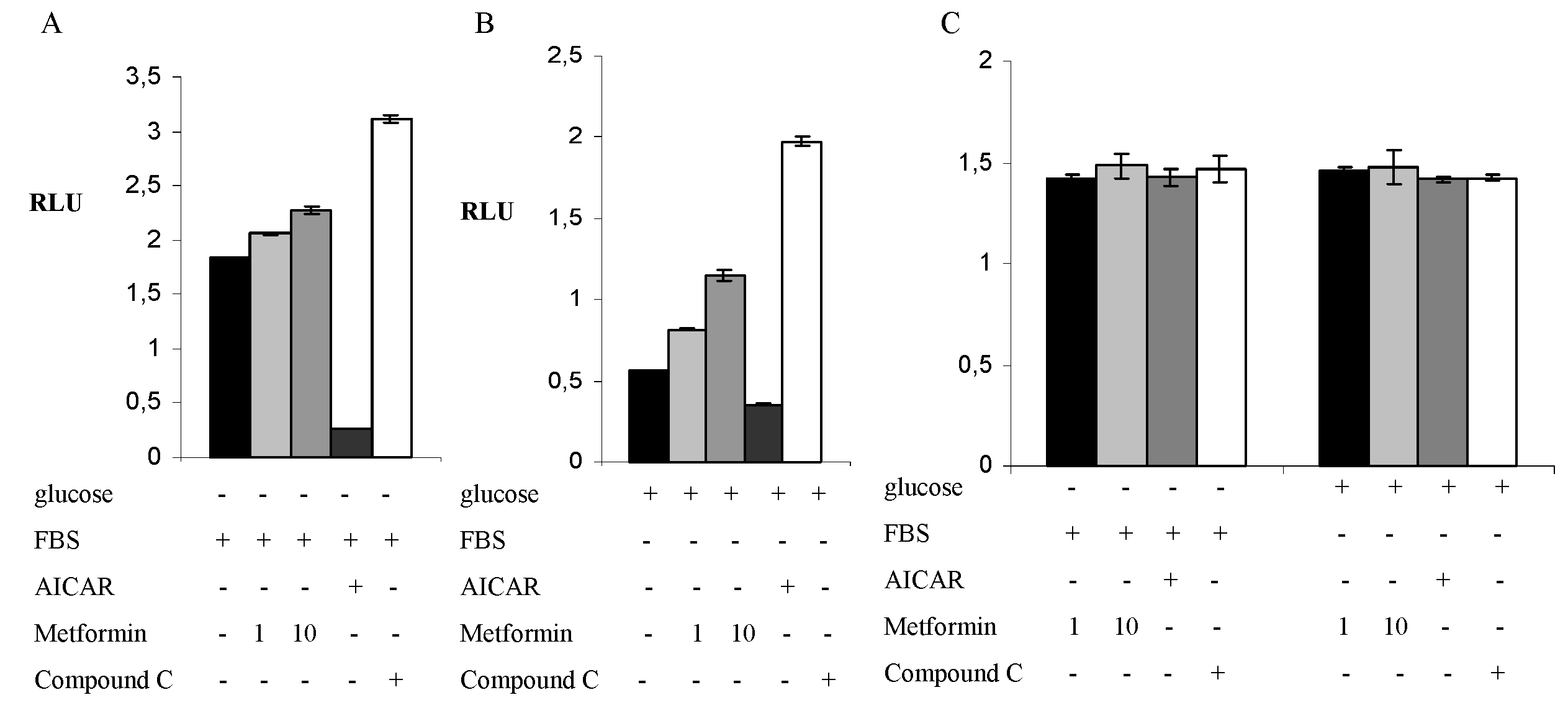

Supplement: Figure S4 — CPT1C 5′UTR repression is relieved by AMPK inhibition. Relative luciferase activity in SV40-FHAS cells transfected with uORF start reporter construct after maintenance in media containing either (A) FBS but no glucose or glucose but no FBS in the presence of AMPK-inhibitors metformin or Compound C, or in the presence of the AMPK-activator AICAR. (B) Relative luciferase activity in cells and transfected with uORF no start reporter construct after maintenance in the presence of AMPK-activators metformin or Compound C, the AMPK-inhibitor AICAR. RLU, Relative Luciferase Units. (TIF) [file pone.0021486.s004.tif]

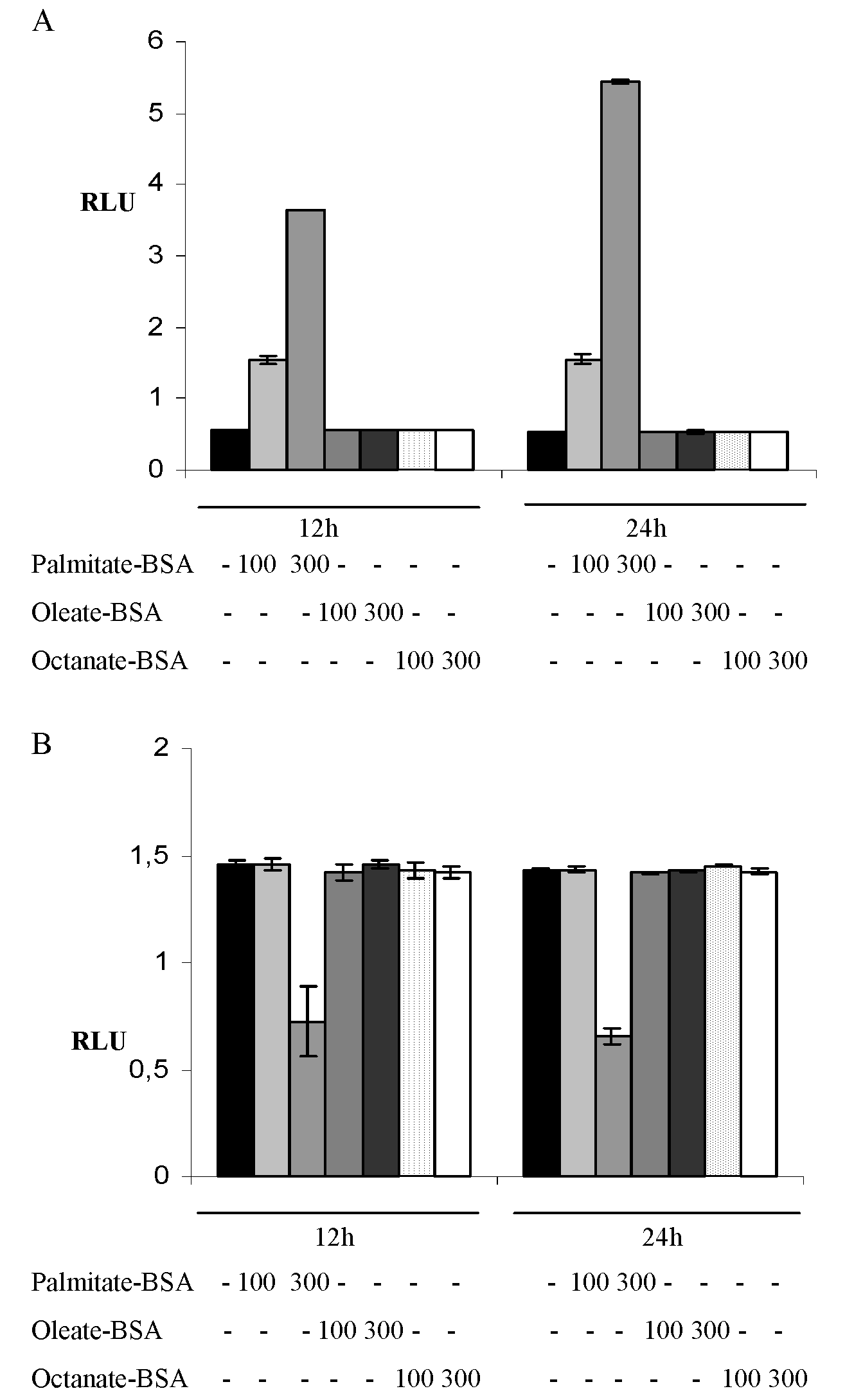

Supplement: Figure S5 — Palmitate relieves CPT1C 5′UTR-mediated translational repression. Relative luciferase activity of (A) uORF start and (B) uORF no start reporter constructs after transfection into SV40-FHAS cells upon treatment with stated BSA conjugated fatty acids for 12 h or 24 h. RLU, Relative luciferase units, Palmitate-BSA, Palmitic acid conjugated BSA, Oleate-BSA, Oleic acid conjugated BSA, Octanate-BSA, Octanoic acid-conjugated BSA. RLU, Relative Luciferase Units. (TIF) [file pone.0021486.s005.tif]
