# Supplementary material for: The CPT1C 5′UTR Contains a Repressing Upstream Open Reading Frame That Is Regulated by Cellular Energy Availability and AMPK
Source: PLoS One. 2011 Sep 22;6(9):e21486. doi: 10.1371/journal.pone.0021486 (PMC3178533; doi:10.1371/journal.pone.0021486)
Supplement: Table S1 — siRNA sequences. Sequences of the shRNAs used for transient AMPK knock down and the scarmbeled control shRNA that was used as a nonspecific control. (DOC) [file pone.0021486.s006.doc]

# Supplementary Figures

**Supplementary Table 1**: **siRNA sequences**

| gene | shRNA sequence (5`-`3) |
| --- | --- |
| Scrambled control | AGGUAGUGUAAUCGCCUUGtt |
| AMPK 1 | GGUUGGCAAACAUGAAUUGtt |
| AMPK 2 | UUCCUUCGCACACGCAAAUAAUAGGtt |
